# Supplementary material for: K-th Nearest Neighbor (KNN) Entropy Estimates of Complexity and Integration from Ongoing and Stimulus-Evoked Electroencephalographic (EEG) Recordings of the Human Brain
Source: Entropy (Basel). 2019 Jan 13;21(1):61. doi: 10.3390/e21010061 (PMC7514170; doi:10.3390/e21010061)
Supplement: Supplementary file 1 [file entropy-21-00061-s001.pdf]

entropy

MDPI

Article

K-Nearest Neighbor (KNN) Entropy Estimates of Complexity and Integration from Non-Stationary Electroencephalographic (EEG) Recordings of the Human Brain

Logan T. Trujillo

Department of Psychology, Texas State University; San Marcos, Texas, 78666, USA; logant@txstate.edu; Tel.: +01-512-245-3623

Supplementary Material

1.1 Categorization Task Stimuli Spatial Frequencies and Orientations

Table S1. Spatial frequencies and orientations for 1-Exemplar Task Gabor stimuli

Category Stimulus	Category 1		Category 2	
	Frequency	Orientation	Frequency	Orientation
Task Order 1	3.86	57.50	3.24	68.30
Task Order 2	1.62	57.50	2.23	68.30
Task Order 3	3.24	21.70	3.86	32.43
Task Order 4	2.24	21.70	1.62	32.43
Task Order 5	3.24	57.50	3.86	68.30
Task Order 6	2.24	57.50	1.62	68.30
Task Order 7	3.86	21.70	3.24	32.43
Task Order 8	1.62	21.70	2.24	32.43

Spatial frequency values are in cycles/° and orientation values are in degrees from vertical.

Table S2. Spatial frequencies and orientations for 2-Exemplar Task Gabor stimuli

	Category Stimulus	Category 1		Category 2	
		Frequency	Orientation	Frequency	Orientation
Task Order 1	*1*	1.49	18.07	2.33	18.07
	2	2.33	36.01	1.49	36.01
Task Order 2	*1*	3.14	18.07	3.97	18.07
	2	3.97	36.01	3.14	36.01
Task Order 3	*1*	1.49	53.95	2.33	53.95
	2	2.33	71.89	1.49	71.89
Task Order 4	*1*	3.14	53.95	3.97	53.95
	2	3.97	71.89	3.14	71.89

Spatial frequency values are in cycles/° and orientation values are in degrees from vertical.

1.2 Distributional Testing of EEG Property Measures

Parametric statistical analyses such as ANOVA typically assume that data is normally distributed. In order to check this assumption, Jarque-Bera tests of univariate normality [67] were performed for each EEG and behavioral measure across subjects. Given the large number of tests, Type-I error was minimized by correcting the p-values for multiple comparisons using the Holm-Bonferroni procedure [49]. None of the EEG measures were significantly different from a

normal distribution (ps > 0.14). (It should be noted, however, that even though the final EEG integration and complexity values entering into the ANOVA were normally-distributed across subjects, the EEG data from which they were computed were non-normal; see Supplementary Materials Section 1.3., below. This suggests that the operations involved in the computation of *I(X)* and $C_I(X)$ smooth the data in accordance with the Central Limit Theorem). Any concern that this approach was overly conservative and thus might lead to Type-II error in this analysis (i.e. some of the measures were non-normal, but did not depart from normality enough to yield a significant test) may be mitigated by the fact that ANOVAs and GEEs are fairly robust to minor violations of distributional assumptions [45-47].

The distributions of the behavioral reaction time data were also not significantly different from normal for both the 1-Exemplar and 2-Exemplar Categorization tasks. However, while the 2-Exemplar task accuracy rates were normally-distributed, the 1-Exemplar accuracy rates were not; this is because accuracy in this task was near ceiling (see Section 3.3 of the main text). This was accounted for in two ways: first, a nonparametric Wilcoxon signed rank test was used to assess accuracy differences between the two categorization tasks. Second, for GEE-based regressions relating accuracy to EEG integration and complexity, accuracy was treated as the independent variable and the EEG measures as the dependent variable; dependent variables are model-dependent in GEE analyses, whereas independent variable are model-independent [52]. Moreover, the GEE analyses used a robust covariance estimator, which allowed for a model-free estimate of the data covariance structure [53].

1.3 Distributional Testing and Gaussian-Transformation of EEG Data

In order to examine how KNN-based entropy estimation of *I(X)* and $C_I(X)$ compared to the Gaussian-based estimation used by previous studies (Section 3.5 of the main text) it was first necessary to assess to what degree the statistical distribution of the dimension-reduced EEG data deviated from normality, then perform a Gaussian-transformation of the data, and finally assess whether or not the data transformation was successful. Following [11], the univariate and multivariate normality of the EEG signals was assessed via Jarque-Bera tests [67] and Royston's Test of multivariate normality [68], respectively, for each trial, condition, and participant. Royston's Test was computed via a publically available MATLAB script [69]. All tests were conducted at the $p < 0.05$, two-tailed, corrected-level. Gaussian transformation of the data was achieved using a previously established method that has been successfully used before with EEG data [11, 66]. The EEG data was transformed on a trial-by-trial basis for each separate condition, and participant. The distributional testing and Gaussian transformation were performed on the observed and simulated EEG data.

Although the distributions of less than 1% of EEG signals on average violated the univariate normality assumption on any given trial prior to Gaussian-transformation, the multivariate normality assumption was violated on 80% of trials on average. After the Gaussian transformation, none of the EEG trials violated univariate or multivariate normality (all ps = 1).

1.4 Gaussian-Based Entropy Estimation

Marginal and joint entropies of the EEG signals were computed via explicit analytic expressions based on the assumption that a set *X* of N_s EEG signals realize continuous univariate and multivariate Gaussian processes with variances σ^2_{ii} and covariance matrix *K* [6,17,70,71]:

$$H(X_i) = \frac{1}{2\ln(2)} \cdot \ln(2\pi e \sigma_{ii}^2) \ , \tag{S1}$$

$$H(X) = \frac{1}{2\ln(2)} \cdot \ln\left\{(2\pi e)^{N_s} |K|\right\} \ . \tag{S2}$$

Conditional entropy was computed according to Equation 3 of the main text. All entropy functions were computed with a correction for any bias that may arise due to the estimation of the covariance

matrices from limited data [70, 72-74]. All entropies were computed in terms of binary units (bits) of information.

Tables S3 – S7 display the quantitative and statistical results of the Gaussian-based computation of *I(X)* and $C_I(X)$. The general pattern and interpretation of these findings are discussed in the main text Sections 3.5, 4.2, and 4.3.

Table S3. Mean KNN estimator-based EEG integration and complexity of Gaussian-transformed observed data

**Induced EEG**		**Observed Data**		**Surrogate Data**	
**Task**	**Condition**	**I(X)**	**C_I(X)**	**I(X)**	**C_I(X)**
1-Exemplar Task	*Prestimulus*	69.44 (0.36)	82.83 (0.08)	57.82 (57.18, 58.46)	86.69 (86.30, 87.08)
	Poststimulus	68.98 (0.42)	82.97 (0.05)	57.30 (56.71, 57.88)	86.57 (86.15, 86.98)
2-Exemplar Task	*Prestimulus*	69.43 (0.42)	82.88 (0.07)	57.88 (57.03, 58.72)	86.65 (86.22, 87.08)
	Poststimulus	68.71 (0.41)	82.89 (0.06)	57.12 (56.48, 57.76)	86.46 (86.06, 87.86)
Resting Task	*Eyes Open*	70.70 (0.41)	82.10 (0.04)	58.31 (57.65, 58.97)	86.50 (85.98, 87.02)
	Eyes Closed	73.02 (0.46)	82.21 (0.05)	60.19 (59.32, 61.05)	86.40 (85.97, 86.83)
**Evoked EEG**					
**Task**	**Condition**				
1-Exemplar Task	*Prestimulus*	66.13 (0.69)	79.45 (0.86)	57.81 (57.20, 58.42)	86.73 (86.37, 87.09)
	Poststimulus	79.20 (1.35)	82.72 (0.84)	57.13 (56.59, 57.68)	86.43 (86.13, 86.73)
2-Exemplar Task	*Prestimulus*	65.58 (0.64)	80.50 (0.85)	57.88 (57.04, 58.72)	86.74 (86.30, 87.19)
	Poststimulus	81.61 (1.05)	84.84 (0.60)	57.16 (56.56, 57.76)	86.59 (86.01, 87.17)

Note: All values are in bits; SE in parentheses for observed data, 95% CIs in parentheses for surrogate data.

Table S4. Analysis of variance (ANOVA) results for KNN estimator-based EEG integration and complexity of Gaussian-transformed observed data

Task	EEG Measure	Effect	F	P	η^2_P
**Induced EEG**					
Categorization	I(X)	Task	0.57	0.461	0.04
		TI	21.87	0.001	0.59
		Task x TI	6.27	0.024	0.30
	C_I(X)	Task	0.06	0.815	0.01
		TI	1.15	0.301	0.07
		Task x TI	1.57	0.230	0.10
Resting State	I(X)	RS	35.50	0.001	0.70
	C_I(X)	RS	7.15	0.017	0.32
**Evoked EEG**					
Categorization	I(X)	Task	1.42	0.252	0.09
		TI	193.36	0.001	0.93
		Task x TI	10.56	0.005	0.41
	C_I(X)	Task	3.83	0.069	0.20
		TI	17.95	0.001	0.55
		Task x TI	0.81	0.382	0.05

ANOVA factor labels: Task, Behavioral Task; TI, Time Interval; RS, Resting State. All dfs = 1, 15.

Table S5. Mean Gaussian estimator-based EEG integration and complexity of Gaussian-transformed observed data

**Induced EEG**		**Observed Data**		**Surrogate Data**	
**Task**	**Condition**	**I(X)**	**C_I(X)**	**I(X)**	**C_I(X)**
1-Exemplar Task	*Prestimulus*	72.96<br>(0.31)	40.46<br>(0.22)	56.70<br>(55.35, 58.06)	47.95<br>(46.87, 49.03)
	Poststimulus	72.11<br>(0.29)	40.85<br>(0.18)	55.90<br>(54.63, 57.17)	47.60<br>(46.50, 48.70)
2-Exemplar Task	*Prestimulus*	73.00<br>(0.34)	40.46<br>(0.24)	56.85<br>(55.24, 58.48)	47.98<br>(46.85, 49.11)
	Poststimulus	72.01<br>(0.31)	40.87<br>(0.18)	55.62<br>(54.30, 56.93)	47.44<br>(46.34, 48.53)
Resting Task	*Eyes Open*	76.93<br>(0.26)	39.20<br>(0.17)	58.29<br>(57.07, 59.50)	48.54<br>(47.28, 49.80)
	Eyes Closed	78.23<br>(0.34)	38.36<br>(0.21)	60.68<br>(59.24, 62.11)	48.29<br>(47.33, 49.25)
**Evoked EEG**					
**Task**	**Condition**				
1-Exemplar Task	*Prestimulus*	70.72<br>(0.81)	40.15<br>(0.65)	57.65<br>(56.42, 58.89)	45.45<br>(44.64, 46.26)
	Poststimulus	93.32<br>(2.46)	46.10<br>(0.81)	72.79<br>(68.00, 77.58)	49.41<br>(48.06, 50.76)
2-Exemplar Task	*Prestimulus*	70.51<br>(1.04)	40.04<br>(0.69)	57.46<br>(56.03, 58.90)	45.66<br>(44.65, 46.67)
	Poststimulus	96.53<br>(1.98)	47.65<br>(0.59)	75.10<br>(71.00, 79.19)	50.28<br>(49.15, 51.41)

Note: All values are in bits; SE in parentheses for observed data, 95% CIs in parentheses for surrogate data.

Table S6. Analysis of variance (ANOVA) results for Gaussian estimator-based EEG integration and complexity of Gaussian-transformed observed data

**Induced EEG**					
**Task**	**EEG Measure**	**Effect**	**F**	**P**	**η^2_P**
Categorization	I(X)	Task	0.07	0.795	0.01
		TI	70.34	0.001	0.82
		Task x TI	2.32	0.149	0.13
	C_I(X)	Task	0.01	0.953	0.0
		TI	17.43	0.001	0.54
		Task x TI	0.03	0.859	0.01
Resting State	I(X)	RS	27.33	0.001	0.65
	C_I(X)	RS	45.10	0.001	0.75
**Evoked EEG**					
Categorization	I(X)	Task	1.62	0.222	0.10
		TI	138.25	0.001	0.90
		Task x TI	3.29	0.090	0.18
	C_I(X)	Task	3.51	0.081	0.19
		TI	156.86	0.001	0.91
		Task x TI	9.77	0.007	0.40

ANOVA factor labels: Task, Behavioral Task; TI, Time Interval; RS, Resting State. All dfs = 1, 15.

Table S7. Mean KNN estimator-based and Gaussian estimator-based EEG integration and complexity of Gaussian-transformed dipole simulation data

**Data Condition**	**Prestimulus**	**Poststimulus**	**F**	**p**	**η^2_P**
**KNN-Based**					
$I(X)_{Induced}$	68.48 (0.02)	69.19 (0.06)	161.3	0.001	0.92
$C_I(X)_{Induced}$	82.04 (0.08)	82.72 (0.10)	19.72	0.001	0.57
$I(X)_{Evoked}$	64.25 (0.62)	73.59 (0.46)	119.11	0.001	0.89
$C_I(X)_{Evoked}$	80.58 (0.60)	83.48 (0.82)	10.19	0.006	0.40
**Gaussian-Based**					
$I(X)_{Induced}$	73.31 (0.03)	71.81 (0.04)	1461.14	0.001	0.99
$C_I(X)_{Induced}$	40.28 (0.01)	40.87 (0.03)	438.93	0.001	0.97
$I(X)_{Evoked}$	69.33 (1.14)	81.50 (0.71)	76.17	0.001	0.84
$C_I(X)_{Evoked}$	37.97 (0.35)	40.60 (0.31)	54.07	0.001	0.78

Complexity and integration values are in bits, GFP values are in μV, order parameters are dimensionless; SE in parentheses. ANOVA parameters describe the significance of prestimulus versus poststimulus differences.

1.5 Basic EEG Dipole Simulations

The development of the dipole simulations of the empirically-observed categorization task data involved the initial exploration of EEG signals that in terms of dipole source amplitude, phase, and changes in synchronization of dipole oscillations and amplitude over time. Each simulation consisted of the creation of one hundred 2 second trials of simple, wide-range (4 – 13 Hz) oscillatory waveforms that varied in terms of initial amplitude, initial synchronization of the waveform phase, and changes in amplitude and phase coupling. General simulation procedures are described in Section 2.9 of the main text, with manipulation of the following parameters: initial amplitude (high: 60 μA-cm, low: 30 μA-cm), starting phase (random: 2π, synched: π/50), Kuramoto parameter K (0, 5, 10), amplitude change across time (constant; variable: σ = 250 ms Gaussian envelopes with ± 1000 ms peak latency; synchronized: σ = 250 ms Gaussian envelopes with ± 39 ms peak latency), and the dependency of variable/synchronized amplitude changes(full independence or full dependence). In addition, high and low amplitude versions of two special waveforms were simulated, one with a nonstationary discontinuous phase created via the method of Theiler et al. [58] and a second created as a multivariate normal process with μ = 0 and σ = 1.

Tables S8 – S11 display the quantitative results of these basic EEG dipole simulations. In general, these simulations showed qualitatively that EEG integration increased with increasing oscillatory synchronization and synchronized amplitude changes, but decreased with overall reductions in dipole amplitude. EEG complexity followed the theoretically predicted relationship with integration as given in Figure 1, but was mainly affected by changes in dipole amplitude when the dipole oscillations and/or amplitude changes were unsynchronized. Induced and evoked EEG

Table S8. Basic EEG dipole source simulations: High amplitude, random start phase

Data Condition	I(X)	C_I(X)	$GFP_{Induced}$	GFP_{Evoked}	$\Lambda_{Induced}$	Λ_{Evoked}
K_{param} = 0, Constant Amplitude	126.71	111.78	819.69	86.94	0.192	0.220
K_{param} = 5, Constant Amplitude	132.33	116.86	1271.70	100.91	0.296	0.279
K_{param} = 10, Constant Amplitude	136.90	117.38	1593.98	156.00	0.331	0.319
K_{param} = 0, Variable Independent Amplitude	116.18	117.70	877.85	80.35	0.213	0.201
K_{param} = 5, Variable Independent Amplitude	121.63	120.40	1234.61	100.43	0.277	0.259
K_{param} = 10, Variable Independent Amplitude	130.78	118.98	1604.06	136.03	0.331	0.294
K_{param} = 0, Variable Dependent Amplitude	131.25	109.75	894.95	85.45	0.221	0.238
K_{param} = 5, Variable Dependent Amplitude	132.99	114.98	1300.91	124.26	0.299	0.289
K_{param} = 10, Variable Dependent Amplitude	139.87	116.54	1709.29	152.93	0.333	0.323
K_{param} = 0, Synched Dependent Amplitude	130.59	111.26	804.79	78.96	0.217	0.203
K_{param} = 5, Synched Dependent Amplitude	135.41	114.62	1213.14	85.85	0.291	0.317
K_{param} = 10, Synched Dependent Amplitude	142.83	115.93	1632.33	115.78	0.33	0.327
K_{param} = 0, Variable Amplitude, Nonstationary phase	116.74	117.91	910.25	90.30	0.219	0.217
Multivariate Normal Process (μ = 0, σ = 1)	67.85	102.70	647.87	67.29	0.252	0.263

All values are averages of electrodes, time, and trials. Complexity and integration values are in bits, power values are in μV, order parameter is dimensionless; SE in parentheses.

power were generally larger for synchronized versus unsynchronized dipole oscillations and amplitude changes, which reflects the constructive versus destructive summation of EEG signals as they are volume-conducted through the scalp. However, as for the empirical data, induced EEG power was greater than evoked power. Finally, induced and evoked EEG power were generally larger for synchronized versus unsynchronized dipole oscillations and amplitude changes, but like EEG complexity was mainly affected by changes in dipole amplitude when the dipole oscillations and/or amplitude changes were unsynchronized.

Table S9. Basic EEG dipole source simulations: Low amplitude, random start phase

Data Condition	I(X)	$C_I(X)$	$GFP_{Induced}$	GFP_{Evoked}	$\Lambda_{Induced}$	Λ_{Evoked}
***K_{param}* = 0, Constant Amplitude**	126.71	111.78	416.23	31.81	0.220	0.167
***K_{param}* = 5, Constant Amplitude**	132.33	116.86	614.35	70.41	0.304	0.318
***K_{param}* = 10, Constant Amplitude**	136.90	117.38	812.27	49.84	0.328	0.334
***K_{param}* = 0, Variable Independent Amplitude**	116.18	117.70	448.79	44.51	0.218	0.184
***K_{param}* = 5, Variable Independent Amplitude**	121.63	120.40	624.51	58.63	0.286	0.253
***K_{param}* = 10, Variable Independent Amplitude**	130.78	118.98	812.15	64.55	0.334	0.318
***K_{param}* = 0, Variable Dependent Amplitude**	131.25	109.75	450.85	40.32	0.221	0.186
***K_{param}* = 5, Variable Dependent Amplitude**	132.99	114.98	644.77	47.72	0.294	0.261
***K_{param}* = 10, Variable Dependent Amplitude**	139.87	116.54	857.65	71.65	0.330	0.333
***K_{param}* = 0, Synched Dependent Amplitude**	130.59	111.26	417.81	35.05	0.216	0.222
***K_{param}* = 5, Synched Dependent Amplitude**	135.41	114.62	603.40	51.09	0.290	0.304
***K_{param}* = 10, Synched Dependent Amplitude**	142.83	115.93	816.21	62.57	0.328	0.309
***K_{param}* = 0, Variable Amplitude, Nonstationary phase**	116.74	117.91	449.22	47.41	0.215	0.148
Multivariate Normal Process (μ = 0, σ = 1)	67.85	102.70	325.85	31.12	0.247	0.244

All values are averages of electrodes, time, and trials. Complexity and integration values are in bits, power values are in μV, order parameter is dimensionless; SE in parentheses.

Table S10. Basic EEG dipole source simulations: High amplitude, synchronized start phase

Data Condition	I(X)	$C_I(X)$	$GFP_{Induced}$	GFP_{Evoked}	$\Lambda_{Induced}$	Λ_{Evoked}
K_{param} = 0, Constant Amplitude	126.71	111.78	1948.82	476.19	0.372	0.374
K_{param} = 5, Constant Amplitude	132.33	116.86	1948.40	378.26	0.372	0.373
K_{param} = 10, Constant Amplitude	136.90	117.38	1948.62	427.74	0.372	0.374
K_{param} = 0, Variable Independent Amplitude	116.18	117.70	1910.16	406.21	0.367	0.369
K_{param} = 5, Variable Independent Amplitude	121.63	120.40	1907.12	427.04	0.368	0.372
K_{param} = 10, Variable Independent Amplitude	130.78	118.98	1914.63	397.18	0.368	0.366
K_{param} = 0, Variable Dependent Amplitude	131.25	109.75	2107.94	418.37	0.374	0.374
K_{param} = 5, Variable Dependent Amplitude	132.99	114.98	2115.96	454.54	0.374	0.374
K_{param} = 10, Variable Dependent Amplitude	139.87	116.54	2104.34	465.12	0.374	0.375
K_{param} = 0, Synched Dependent Amplitude	130.59	111.26	1958.32	281.76	0.370	0.374
K_{param} = 5, Synched Dependent Amplitude	135.41	114.62	1952.69	302.75	0.371	0.374
K_{param} = 10, Synched Dependent Amplitude	142.83	115.93	1962.71	225.23	0.371	0.374
K_{param} = 0, Variable Amplitude, Nonstationary phase	116.74	117.91	890.42	80.13	0.227	0.240
Multivariate Normal Process (μ = 0, σ = 1)	67.85	102.70	650.18	62.33	0.250	0.255

All values are averages of electrodes, time, and trials. Complexity and integration values are in bits, power values are in μV, order parameter is dimensionless; SE in parentheses.

Table S11. Basic EEG dipole source simulations: Low amplitude, synchronized start phase

Data Condition	I(X)	$C_I(X)$	$GFP_{Induced}$	GFP_{Evoked}	$\Lambda_{Induced}$	Λ_{Evoked}
K_{param} = 0, Constant Amplitude	126.71	111.78	973.45	237.56	0.370	0.375
K_{param} = 5, Constant Amplitude	132.33	116.86	969.83	256.34	0.371	0.374
K_{param} = 10, Constant Amplitude	136.90	117.38	976.09	220.55	0.371	0.376
K_{param} = 0, Variable Independent Amplitude	116.18	117.70	953.97	218.06	0.367	0.365
K_{param} = 5, Variable Independent Amplitude	121.63	120.40	954.19	223.16	0.367	0.37
K_{param} = 10, Variable Independent Amplitude	130.78	118.98	955.17	188.01	0.366	0.369
K_{param} = 0, Variable Dependent Amplitude	131.25	109.75	1055.30	205.92	0.372	0.374
K_{param} = 5, Variable Dependent Amplitude	132.99	114.98	1054.52	215.45	0.372	0.371
K_{param} = 10, Variable Dependent Amplitude	139.87	116.54	1054.28	224.26	0.372	0.374
K_{param} = 0, Synched Dependent Amplitude	130.59	111.26	976.55	158.35	0.367	0.373
K_{param} = 5, Synched Dependent Amplitude	135.41	114.62	979.88	137.57	0.366	0.374
K_{param} = 10, Synched Dependent Amplitude	142.83	115.93	977.67	152.26	0.367	0.372
K_{param} = 0, Variable Amplitude, Nonstationary phase	116.74	117.91	450.49	41.58	0.208	0.200
Multivariate Normal Process (μ = 0, σ = 1)	67.85	102.70	324.42	30.68	0.247	0.252

All values are averages of electrodes, time, and trials. Complexity and integration values are in bits, power values are in μV, order parameter is dimensionless; SE in parentheses.

References

All references cited in the Supplementary Materials are listed in the References section of the main text.
